# Proinflammatory Cytokine IL-17 Shows a Significant Association with *Helicobacter pylori* Infection and Disease Severity

**DOI:** 10.1155/2017/6265150

**Published:** 2017-12-17

**Authors:** Piyumali Sandareka Arachchi, Neluka Fernando, Manjula Manoji Weerasekera, Bimalka Senevirathna, Deepaka D. Weerasekera, Chinthika Prabhashinie Gunasekara

**Affiliations:** ^1^Department of Microbiology, Faculty of Medical Sciences, University of Sri Jayewardenepura, Gangodawila, Nugegoda, Sri Lanka; ^2^Department of Pathology, Faculty of Medical Sciences, University of Sri Jayewardenepura, Gangodawila, Nugegoda, Sri Lanka; ^3^Department of Surgery, Faculty of Medical Sciences, University of Sri Jayewardenepura, Gangodawila, Nugegoda, Sri Lanka

## Abstract

**Background:**

The pro- and anti-inflammatory cytokines play an important role in the immune response against *H. pylori* infection. The proinflammatory cytokines of Th17 cells have been suggested to play a major role in *H. pylori* infection and resulting gastric inflammation.

**Objective:**

The objective of this study was to compare the expression of selected inflammatory cytokines (IL-10, IL-17, IL-21, IL-23, and TNF-*α*) in *H. pylori*-infected patients and healthy controls and to understand their association with *H. pylori* infection and disease severity.

**Results:**

The expression levels of IL-17 and IL-23 were significantly higher in *H. pylori-*infected patients. The expression of IL-21 was also higher in *H. pylori-*positive patients but there was no significant association with infection. IL-17 expression showed a significant increase with the severity of chronic gastritis.

**Conclusion:**

The proinflammatory cytokine, IL-17, shows a significant association with *H. pylori* infection and disease severity in a Sri Lankan dyspeptic patient population.

## 1. Introduction

*Helicobacter pylori* (*H. pylori*) infects and colonizes the gastric epithelium of humans, resulting in gastric disease of varying severity [1]. The immunopathogenesis of *Helicobacter pylori* infection is still an enigma. Host and bacterial factors play an important role in the immunopathogenesis [2–4]. The bacterium induces both innate and adaptive immune responses in local gastric environment as well as a heterogeneous systemic IgG response [5]. Many studies have described the development of *H. pylori*-induced gastritis as predominant, due to Th1 cells, while Th2 cells have a protective effect [6]. However, the Th1 response alone is insufficient to explain the pathogenesis of *H. pylori-*induced gastritis.

A role of Th17 cells in the pathogenesis of gastritis has been suggested by several groups [7]. Th17 cells are recently described to be important in immunity to extracellular bacterial and fungal infections [8]. The cytokine profile of Th17 cells includes IL-17A, IL-17F, IL-21, and IL-22. The expansion of the Th17 cells is promoted by IL-23 and IL-21 which is responsible for maintenance of the Th-17 cell population [9].

The immunopathogenic role of Th-17 cells has been described with regard to autoimmune diseases such as systemic lupus erythematosus (SLE) and Sjögren syndrome, by promoting chronic inflammation [10, 11]. Further, Th17 cells have shown to be protective in colitis [12]. Studies done using *H. pylori-*infected biopsies report the upregulation of IL-17 at both RNA and protein levels compared to uninfected biopsies [13]. Further, the local IL-17 levels in the gastric mucosa at the infection site have been shown to have high IL-17 levels [14]. The cytokine IL-17 is suggested to play a decisive role in the neutrophil recruitment in *H. pylori-*infected gastric mucosa and stimulates the fibroblasts to produce matrix metalloproteinases which further contributes to mucosal damage. Recently, the reports on the association of Th17 cells with *H. pylori* infection have emerged but the exact role of Th17 cells in *H. pylori* infection is yet unclear.

## 2. Materials and Methods

Twenty *H. pylori*-positive patients (confirmed by histology) and 30 age sex-matched healthy control group were included in the study. Ethical approval was granted from the Ethics Review Committee, University of Sri Jayewardenepura (14/15) and Colombo South Teaching Hospital, Sri Lanka. The control group consisted of healthy individuals negative for *H. pylori* IgG (Bioactiva Diagnostica, GmBH), who did not have any symptoms of dyspepsia and were not under any health conditions requiring medical attention. Gastritis was staged according to the modified Sydney system in Hematoxylin & Eosin-stained biopsies. A blood specimen of 5 mL was collected from each patient and healthy individual for the ELISA procedure. The blood specimen was centrifuged, serum separated, and stored at −80°C.

### 2.1. Enzyme-Linked Immunosorbent Assay for Detection of Serum Cytokines and *H. pylori* IgG

The expression of selected cytokines (IL-10, IL-17, IL-21, IL-23, and TNF-*α*) in serum specimens were determined using enzyme-linked immunosorbent assay (Mabtech, Sweden). The healthy controls were selected using a *H. pylori* IgG assay (Bioactiva Diagnostica, GmBH). Healthy patients positive for *H. pylori* IgG were excluded from the study.

*H. pylori* IgG ELISA was carried out to determine the serological status of the healthy volunteers following the manufacturer's instructions (Bioactiva Diagnostica, GmBH). Briefly, precoated ELISA plates were incubated with serum specimens diluted according to the manufacturers' instructions. After incubating at 37°C for 1 hour, the plate was washed with washing buffer and conjugate solution was added. The plate was incubated at 20–25°C for 30 minutes before washing. The substrate solution was added to the plate and incubated at 20–25°C for 15 min.

For the determination of cytokines, the sterile 96-well plate was coated with coating antibody on the first day and incubated at 4–8°C overnight. The coated plate was washed with 200 *μ*L of PBS solution twice (pH 7.4). Incubation buffer (PBS + 0.05% Tween 20 + 1% BSA) 200 *μ*L was added to the wells (except blank) and allowed to incubate at room temperature. After 1 hour, the plate was washed with washing buffer five times.

Serial dilution of cytokine standards was prepared in the range instructed by the manufacturer and added to the wells (each standard was added to 2 wells to provide duplicates). The serum specimens were diluted 1 : 1 with ELISA diluent and added to the wells in duplicate. The wells for blank control were left empty. The plate was allowed to incubate for 2 hours at room temperature. The plate was washed 5 times using wash buffer (PBS + 0.05% Tween 20).

The secondary antibody was added to the wells and the plate was incubated for 1 hour. The plate was washed 5 times and 100 *μ*L of streptavidin-HRP (diluted 1 : 1000 in incubation buffer) was added to each well. After incubating for 1 hour, the plate was again washed 5 times. 100 *μ*L of TMB substrate was added to each well, and the plate was covered with aluminum foil and incubated at room temperature for 40 min. At the end of the incubation period, 100 *μ*L of stop solution (1 M H_2_SO_4_ solution) was added to each well prior to absorbance measurement.

Absorbance of all ELISAs was measured at 450 nm using MPSCREEN MR-96A ELISA reader.

### 2.2. Data Analysis and Statistical Calculations

Corrected absorbance values (AbsC) were calculated by subtracting the mean absorbance value of blank sample (AbsB) from the mean absorbance value of the test sample (AbsT).

(1) $$\begin{array}{c} AbsC=AbsT–AbsB. \end{array}$$

Corrected absorbance values of all standards, patient samples, and healthy controls were calculated. Log 10 values of all unknown sample (test) concentrations were interpolated using a sigmoidal dose-response curve with GraphPad Prism version 7 (GraphPad Software Inc). Antilog values were calculated to determine the unknown sample concentration.

The cytokine expression values in patients and healthy controls were entered in a new data sheet in GraphPad Prism. The significance between *H. pylori* patients and healthy controls was calculated using Mann–Whitney *U* test using 95% confidence interval. The significant association of cytokine expression with disease severity was calculated using the Kruskal-Wallis test.

## 3. Results

The *H. pylori*-positive dyspeptic patient group consisted of 20 patients aged 18–70 years. Thirteen were male and 7 were female. The control group (*H. pylori*-negative, healthy) consisted of 18 males and 12 females with ages ranging from 20 to 65 years of age. Of the 20 *H. pylori*-infected patients, 13 had mild chronic gastritis while 6 had moderate gastritis and only 1 patient had severe gastritis.

Expression of serum cytokines (IL-17A, IL-21, IL-23, IL-10, and TNF-*α*) in *H. pylori*-infected patients was compared with healthy controls and correlated with disease severity (mild chronic gastritis, moderate chronic gastritis, and severe chronic gastritis).

**Figure 1:**
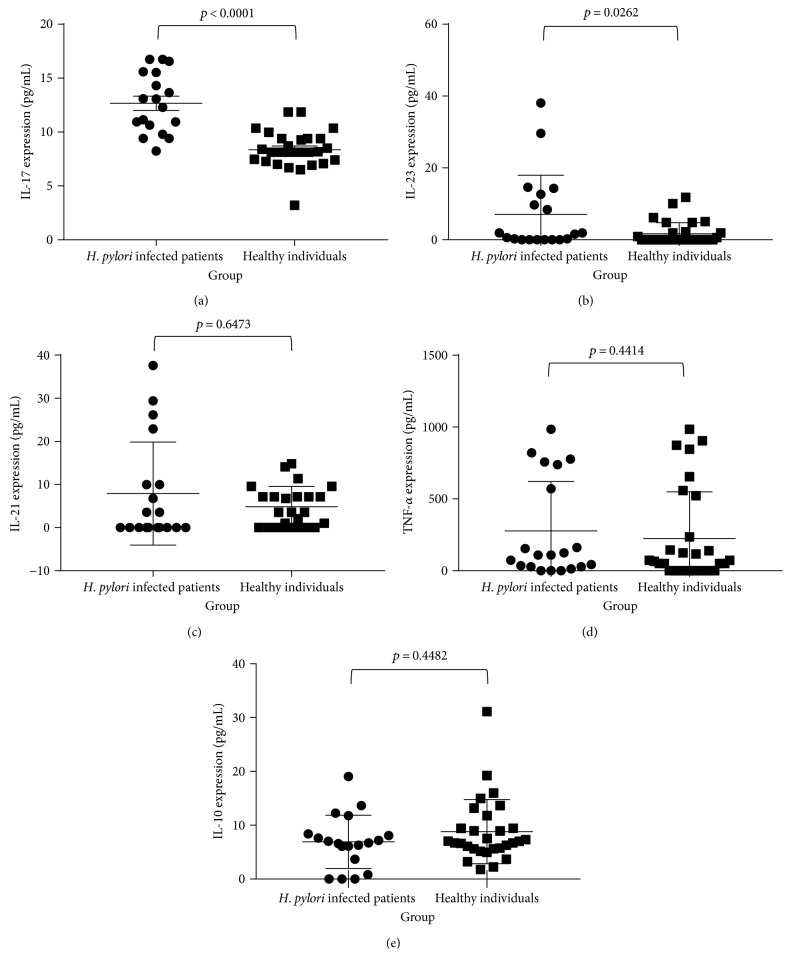
Comparison of IL-17, IL-23, IL-21, TNF-*α*, and IL-10 levels (pg/mL) of *H. pylori-*infected patients and healthy individuals. Expression levels of selected cytokines were compared between *H. pylori*-infected patients (*n* = 20) and healthy controls (*n* = 30). Association of cytokine expression with *H. pylori* infection was calculated using the Mann–Whitney *U* test. (a) Comparison of IL-17 concentration among the two groups (*p* < 0.0001). (b) Comparison of IL-23 expression among the two groups (*p* = 0.0262). (c) Comparison of IL-21 expression (*p* = 0.6473). (d) TNF-*α* expression levels among *H. pylori*-infected patients and healthy controls (*p* = 0.4414). (e) Comparison of IL-10 concentration among the two groups (*p* = 0.4482).

On comparison, the association between *H. pylori* infection and cytokine expression levels, IL-17A (*p* < 0.0001) and IL-23 (*p* = 0.0262) were significantly elevated in the patients with confirmed *H. pylori* infection (Figures 1(a) and 1(b)). Further, it was observed that IL-21 and TNF-*α* levels were elevated in this group although not statistically significant (Figures 1(c) and 1(d)).  Table 1 depicts the mean and the range of cytokine concentrations for *H. pylori-*positive patients and healthy controls. The mean cytokine concentrations observed for IL-17A and IL-23 for *H. pylori*-positive group were 12.67 pg/mL and 7.06 pg/mL, respectively, while in healthy individuals, the mean concentrations were 8.37 pg/mL and 1.68 pg/mL, respectively.

**Figure 2:**
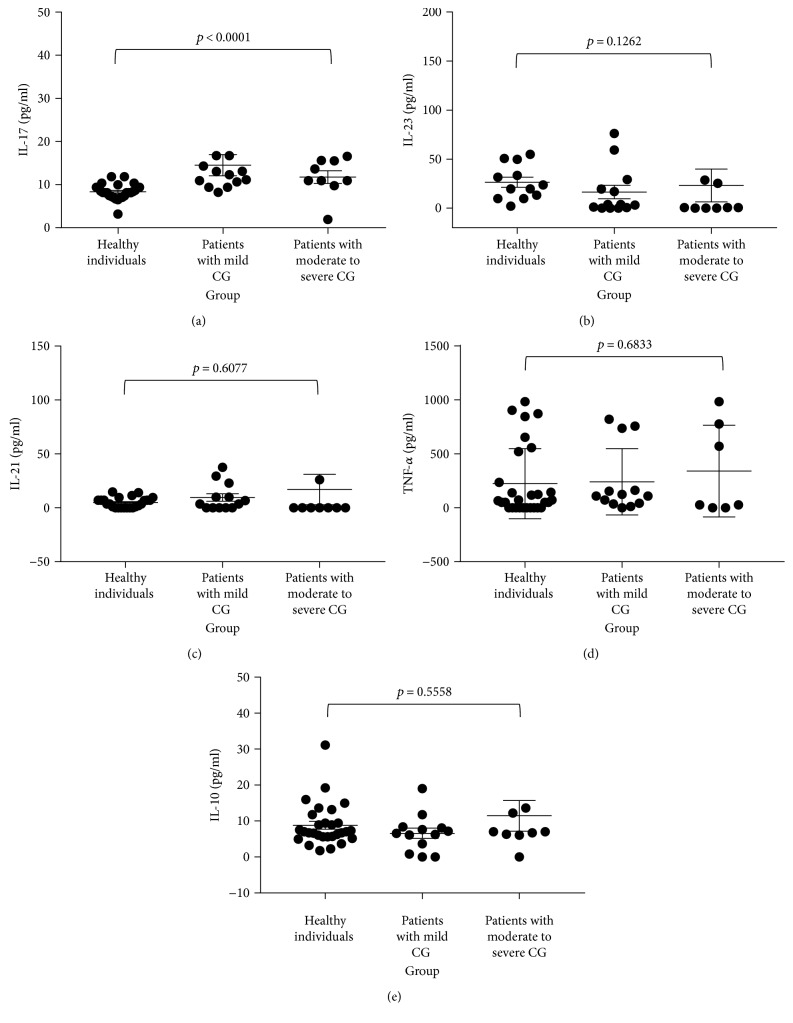
Correlation of cytokine expression in *H. pylori*-infected patients with disease severity. The expression of cytokines in healthy individuals, *H. pylori*-infected patients with mild chronic gastritis (CG), and *H. pylori*-infected patients with moderate to severe chronic gastritis was compared using the Kruskal-Wallis test. (a) Comparison of IL-17 expression (*p* < 0.0001). (b) Comparison of IL-23 expression (*p* = 0.1262). (c) Comparison of IL-21 expression (*p* = 0.6077). (d) Comparison of TNF-*α* expression (*p* = 0.6833). (e) Comparison of IL-10 expression (*p* = 0.5558).

Serum IL-17A concentration was statistically significant between healthy controls and patients with mild chronic gastritis (*p* = 0.0001) and with moderate to severe chronic gastritis (*p* = 0.0136). The expression of cytokines IL-23, IL-21, TNF-*α*, and IL-10 did not have a significant association with the severity of chronic gastritis in this study (Figure 2, Table 2).

## 4. Discussion

The serum cytokine expressions of *H. pylori*-positive dyspeptic patients show a significant elevation of IL-17A and IL-23. Further, raised levels of IL-21 among *H. pylori*-positive patients suggest a possible role of Th17 cells in the immunopathogenesis of *H. pylori*-associated gastritis.

*H. pylori* infect the gastric mucosa, and hence, it can induce local as well as systemic immune response in the host. The mucosal cytokine expression in *H. pylori* infection has provided strong evidence for the inflammatory response mediated by *H. pylori* in the local mucosa [13, 15]. However, serum cytokine response to *H. pylori* infection needs elucidation as the infection can also produce systemic immune responses in the host [16–18]. In a study by Serelli-Lee et al., the circulating Th17 cells were elevated in patients with active *H. pylori* infection and significantly elevated in patients with past *H. pylori* infection compared to the healthy group. Their findings indicate elevation of Th17 cells in the gastric mucosa as well as in the blood indicating that serum cytokine levels can reflect the Th17 response in *H. pylori* infection [14]. Further, this is supported by studies reporting elevation of Th17-associated cytokines in the serum in *H. pylori-*infected patients [16]. In the present study, it was not possible to obtain mucosal biopsies from healthy individuals; therefore, the Th17-associated cytokine levels of serum was investigated.

Th17 cells are increasingly being associated with immunity to extracellular bacterial and fungal infections. Recent studies provide evidence for presence of Th17 cells in *H. pylori*-infected gastric biopsies and elevation of proinflammatory cytokines including IL-17 compared to healthy controls similar to our observations [7, 19]. Subsequently, Serelli-Lee et al. reported that Th17 cells persist even after *H. pylori* eradication resulting in persistence of an IL-17A response that may contribute to pathogenesis of gastric cancer from the early stages of *H. pylori* infection [14]. Serelli-Lee et al. hypothesized that chronic IL-17A signaling and coexpression of IL-22 may drive persistent expression of antimicrobial peptides and matrix metalloproteinases in the gastric mucosa promoting carcinogenesis.

IL-17A secretion by Th17 cell is regulated by IL-23 through a STAT3-dependent pathway [20]. IL-23 is secreted by several immune cells including dendritic cells, macrophages, and neutrophils in the gastric mucosa. Elevated IL-23 levels in the *H. pylori*-infected gastric biopsies have been reported by Koussoulas in 2008 [21]. Horvath et al. reported a positive correlation between levels of IL-23 and the infiltration levels of neutrophils and monocytes in patients with *H. pylori* infection [22]. It is suggested that IL-23 plays a role in the activation of the immune response and induction of gastritis in response to *H. pylori*.

The expression levels of TNF-*α* was relatively high in all *H. pylori-*positive patients, and a high expression of TNF-*α* was observed in patients with mild chronic gastritis and moderate to severe chronic gastritis compared to controls, even if there was no significant difference. IL-17 can stimulate TNF-*α* secretion along with induction of other proinflammatory cytokines IL-1, IL-6, and matrix metalloproteinases from both immune and nonimmune cells [23]. The elevation of TNF-*α* seen in our study can be explained by the significantly elevated IL-17 seen in *H. pylori*-positive patients compared with the healthy controls. TNF-*α* is a proinflammatory cytokine which can act on endothelial cells to stimulate expression of adhesion molecules facilitating the extravasation of neutrophils into the site of infection on the mucosal tissue. It can further activate T cells and stimulate cytokine production by macrophages and monocytes [23]. TNF-*α* has been shown to be associated with increased apoptosis independently of the VacA and CagA status of *H. pylori*. Siregar et al. [18] reported that TNF-*α* expression was significantly associated with both *H. pylori* infection and degree of chronic inflammation [18]. Although not significant, a clear elevation in the mean cytokine expression was observed in moderate to severe gastritis patients compared to mild gastritis patients in our study which further supports the findings of Siregar et al.

In a study conducted in Iran, mucosal IL-21 mRNA levels were quantitatively analyzed using real-time PCR. IL-21 expression was significantly higher in *H. pylori*-infected patients and a significant correlation was observed between IL-21 mRNA levels and chronic inflammation [24]. In our study, the expression of IL-21 was higher in *H. pylori*-infected patients but no significant association could be observed. The elevation of IL-21 among *H. pylori*-infected patients observed in our study and the significant elevation of IL-17 together suggests that in the Th-17 pathway, IL-21 helps to maintain the Th-17 cells and thus regulate Th17 effector responses during chronic *H. pylori* infection.

## 5. Conclusions

In conclusion, the significant elevation of IL-17 and IL-23 and high expression of IL-21 and TNF-*α* in the serum of *H. pylori-*positive patients further supports the role of Th-17 cells in the pathogenesis of *H. pylori* infection and the severity of gastritis.

## Figures and Tables

**Table 1:** Expression of cytokines in serum and their association with *H. pylori* infection.

Cytokine	Healthy group		*H. pylori*-infected group		*p* value
	Range	Mean	Range	Mean	
IL-10	1.77–31.13	8.83	0–19.04	6.91	0.4482
IL-17	3.20–11.86	8.37	8.24–16.74	12.67	<0.0001^∗^
IL-21	Undetectable−14.8	4.86	Undetectable−37.62	7.89	0.6473
IL-23	Undetectable−11.8	1.68	Undetectable−38.08	7.06	0.0262^∗^
TNF-*α*	Undetectable−984.7	224.6	Undetectable−984.7	276.4	0.4414

^∗^ *p* < 0.05.

**Table 2:** Expression of cytokines in serum and their association with disease severity.

Participant group depending on disease severity	Mean expression (pg/mL)				
	IL-10	IL-17	IL-21	IL-23	TNF-*α*
Healthy controls	8.825	8.367	4.861	26.52	224.6
*H. pylori-*infected patients with mild chronic gastritis	6.579	14.52	9.518	16.43	241.6
*H. pylori-*infected patients with moderate to severe chronic gastritis	12.82	12.0	21.86	29.61	341.2
*p* value	0.5558	<0.0001^∗^	0.6077	0.1262	0.6833

^∗^ *p* < 0.05.
